# Neural correlates of altered loss aversion in alcohol use disorder: preliminary evidence of sex-related differences from 18F-FDG-PET imaging

**DOI:** 10.3389/fpsyt.2026.1847372

**Published:** 2026-05-13

**Authors:** Maria Arioli, Irene Bossert, Daniela D’Ambrosio, Elena Maria Andreolli, Giuseppe Trifirò, Nicola Canessa

**Affiliations:** 1 IUSS Cognitive Neuroscience (ICoN) Center, Scuola Universitaria Superiore IUSS, Pavia, Italy; 2 Nuclear Medicine Unit of Pavia Institute, Istituti Clinici Scientifici Maugeri IRCCS, Pavia, Italy; 3 Medical Physics Unit of Pavia Institute, Istituti Clinici Scientifici Maugeri IRCCS, Pavia, Italy; 4 Cognitive Neuroscience Laboratory of Pavia Institute, Istituti Clinici Scientifici Maugeri IRCCS, Pavia, Italy

**Keywords:** alcohol use disorder, decision-making, insula, loss aversion, PET, posterior frontomedial cortex, punishment sensitivity, sex-related differences

## Abstract

Previous findings suggest that the altered punishment sensitivity displayed by individuals with Alcohol Use Disorder (AUD) might reflect lower levels of loss aversion (LA), i.e., the tendency to overweigh negative relative to positive choice consequences. However, whether lower LA represents a core facet of abnormal decision-making in AUD, rather than a secondary consequence of defective executive functioning, remains debated. We used a gambling task to compare LA across 22 AUD patients and 19 age-, sex-, and education-matched healthy controls, and 18F-FDG-PET to investigate its neural correlates in the AUD sample, using 42 age/sex-matched PET healthy controls as the reference group for analyses on brain metabolism. Although AUD patients displayed both significantly lower LA and a hypometabolic pattern in the anterior cingulate and anterior insular nodes of the salience network, the behavioral finding was not explained by altered attentional or executive skills. Instead, we observed a negative relationship with left anterior insular metabolism and LA, possibly reflecting altered regulation of emotions associated with interoceptive processing. Within the AUD sample, lower LA was associated with a steeper negative relationship with frontomedial metabolism in males than in females, suggesting sex-related modulation of its neural correlates. While providing novel insights into the hypometabolic brain pattern associated with lower LA in AUD, these findings unveil sex-specific effects calling for tailored intervention approaches both in research and clinical practice.

## 1 Introduction

Alcohol Use Disorder (AUD) is a pervasive and chronic condition characterized by excessive and compulsive alcohol consumption despite its adverse psychological, physical and social consequences (1), affecting over 29.5 million people worldwide (2). Growing evidence shows that AUD is associated with both structural (3, 4) and functional (5) neural alterations, which may underlie cognitive impairments, particularly in executive functions (6) including behavioral learning (7) and decision-making (8, 9).

A critical hallmark of addictions, including AUD, is indeed the transition from voluntary and reward-based to uncontrolled and compulsive behavior (10). This shift is usually attributed to abnormal action-reward evaluations (11, 12) and poorer learning from negative experiences (13, 14). However, even healthy individuals display considerable individual differences in evaluating the trade-off between positive and negative choice outcomes, reflected in a variable susceptibility to multiple decision-making biases. One such bias is “loss aversion” (LA), i.e., the tendency to overweigh negative outcomes compared with positive ones, and thus to prefer avoiding losses over acquiring equivalent gains (15, 16). Such a cautionary approach departs from the reduced punishment sensitivity observed in addictions, and lower levels of LA in AUD patients (17, 18) might help explain behavioral alterations in this condition. For example, multifaceted evidence relates individual differences in LA with emotion regulation (19), interoception (20) and attentional control (21), which are known to be impaired in addiction (22, 23). Indeed, deficits in interoceptive awareness (24), combined with difficulties in controlling impulsive behaviors and in accessing effective emotion regulation strategies (25), may significantly contribute to the maintenance of drinking behavior (26). These findings support the view that lower LA represents a core aspect, rather than a secondary manifestation of executive impairments, in AUD (27). At the same time, available evidence does not yet allow a definitive conclusion on whether altered LA primarily represents a consequence, a vulnerability factor, or both in relation to alcohol misuse. Although previous studies reported mixed findings concerning the predictive value of decision-making measures for later alcohol outcomes (28), prospective evidence indicates that low LA predicts sustained heavy episodic drinking from age 18 to 24 in young men (29). Moreover, low LA has been associated with higher risk of alcohol and other substance use independently of delay discounting, i.e., a measure of impulsive choice (30). Further evidence is thus needed to clarify the direction of the association between altered LA and AUD, as well as its possible modulators including sex (31).

While LA has been cognitively explained through a “value” function translating objective economic quantities into subjective values (15), functional magnetic resonance imaging (fMRI) studies have shown that, in healthy individuals, the behavioral overweighing of prospective losses relative to gains reflects a pattern of “neural loss aversion” in specific brain structures. Such pattern involves bidirectional responses of activation or deactivation for anticipated gains or losses, with the asymmetry between loss- and gain-related responses tracking individual differences in LA (32–34). This pattern was found in the ventral striatum and midcingulate cortex (more deactivated by anticipated losses than activated by gains; 32, 33, 35), as well as in the insula (more activated by anticipated losses than deactivated by gains; 32). While the functional role of these regions is still debated, their consistent involvement in gain-loss evaluations is in line with our previous evidence that lower LA in AUD patients is not explained by basic executive abilities such as attention or working memory (18) but rather reflects neural mechanisms involving the trade-off between appetitive and aversive motivational drives. In particular, abnormal levels of LA in AUD patients were found associated with both altered resting-state fMRI connectivity from the medial temporal to the insular-opercular cortex, possibly suggesting altered emotional and interoceptive responses to prospective negative outcomes (27; see 36), and GM atrophy in the posterior frontomedial cortex (18), possibly affecting a specific decision-making process such as conflict resolution at the response level (37). Interestingly, these neural mechanisms were also associated with sex differences in susceptibility to psychological processes favoring problem drinking (31).

On this basis, here we aimed to assess the relationship between altered LA and brain functioning in AUD, and to evaluate potential sex-based differences in this relationship. To this purpose, we complemented previous MRI findings with novel evidence from 18F-Fluoro-Deoxy-Glucose Positron Emission Tomography (18F-FDG-PET), which provides a more direct assessment of regional brain metabolism and its relationship with cognitive-behavioral alterations (38, 39). FDG-PET was used to investigate the neural bases of altered punishment sensitivity in 22 AUD patients. Regional metabolism in this sample was compared with that of 42 age-matched PET healthy controls (PET-HCs) drawn from a previously validated database, whereas behavioral LA was compared with 19 age-, sex- and education-matched healthy controls. We predicted that lower LA in AUD patients would be associated with altered brain metabolism in regions previously reported to exhibit both a “neural loss aversion pattern” in healthy individuals (32, 33, 35), and neuro-structural or functional alterations in AUD, such as the insular (27) or posterior frontomedial (18) cortex, with possible modulation by sex.

## 2 Materials and methods

### 2.1 Participants

The sample comprised 22 adult patients diagnosed with AUD (9 females) and 19 healthy control participants (HCs; 8 females), matched on age, sex and education level (**Supplementary Table 1**). There was no significant difference between AUD patients and HCs in these demographic variables, nor between male and female AUD patients in substance use variables (duration of alcohol use, daily dose intake, duration of abstinence before enrollment) or clinical parameters (body-mass-index (BMI), nutritional status, sleep status, anxiety level) (**Supplementary Table 1**). AUD patients were recruited during a 28-day in-patient alcohol withdrawal treatment at the Functional Rehabilitation Unit of ICS Maugeri-Pavia (Italy). They were included in the study after at least 10 days of detoxification, with benzodiazepine treatment being discontinued a minimum of 8 days before PET scanning.

Inclusion criteria for AUD patients were an age range of 20–65 years, and a diagnosis of AUD according to DSM-5 criteria. Their drinking history was assessed with respect to quantity, type and duration of alcohol consumption, that allowed to compute alcohol intake as the average number of daily standard units of alcohol (UA), with one UA = 12 g of ethanol (approximately 330 ml of beer, 125 ml of wine, or 40 ml of hard liquor). Disease duration for patients ranged from 1 to 26 years (mean: 10.40, standard deviation (SD): 6.43). All AUD patients classified as heavy drinkers.

Both patients and HCs were excluded in case of current or past neurological or psychiatric disorders other than AUD, or any comorbid condition other than nicotine dependence. Other exclusion criteria included a family history of neuro-psychiatric disorders, current use of psychotropic substances or medications, past brain injury or loss of consciousness, severe medical conditions, inability to complete the neuro-cognitive assessment, and contraindications to PET. HCs were also screened for alcohol misuse/abuse, using a cut-off of mean alcohol consumption of <2 UAs/day for males and <1 UA/day for females. Additionally, they were asked to abstain from alcohol for at least 10 days prior to the study, and compliance with this requirement was assessed through pre-experiment interviews.

Written informed consent was obtained from all participants, and the study protocol - developed in accordance with the latest version of the Declaration of Helsinki - was approved by the ICS Maugeri Ethics Committee (Pavia, Italy).

### 2.2 Cognitive assessment of executive functions and loss aversion

#### 2.2.1 LA and risk-taking

The collection and analysis of behavioral data have been previously described (18, 27). Briefly, participants’ LA degree was measured through a gambling task entailing the mental anticipation and evaluation of real monetary gains and losses (32, 33, 40). They were presented with 104 mixed gambles, each being associated with a 50% probability of winning or losing different sums of money. The potential gains and losses ranged symmetrically between 1 and 99 (arbitrary units) and were not significantly correlated with each other. In every trial they were asked to choose between the mixed gamble and the status quo (0), after being informed that any accepted gambles would be played for real money, thus leading to increase or decrease an initial monetary endowment. Since performance in this task is also influenced by individual differences in risk attitude (16), the latter was assessed with the Cambridge Gambling Task (CGT; 41) and used to remove its potentially confounding effect from the LA parameter (2.2.2). Results concerning the other CGT measures have been reported elsewhere (42, 43) and will not be further discussed here.

#### 2.2.2 LA estimation

As previously reported (18, 27, 32, 33, 40), we estimated participants’ behavioral LA based on the notion that positive and negative prospective outcomes may be weighted differently (15). A logistic regression was therefore fitted to each participant’s accept/reject decisions (dependent variable), using the magnitude of losses and gains as independent variables to estimate their corresponding “beta” regression coefficients and, finally, a λ parameter defined as their ratio (33). To control for the potential confounding effect of risk attitude, the behavioral LA parameter was residualized against the CGT risk-taking score using a linear regression model fitted across the entire sample (AUD and HC combined), with the λ and CGT “risk taking” parameters as dependent and independent variables, respectively. The resulting residuals were then used in the between-group analyses.

#### 2.2.3 Neuro-cognitive assessment

To investigate the possible influence of executive and working-memory skills on individual variations in behavioral LA (44, 45), participants underwent a neuro-cognitive assessment (46, 47) aimed at evaluating: a) processing speed and attention, through the Trail Making Test (TMT, parts A and B; 48, 49); b) working-memory, with the 10- and 30-second interference memory tests (47); c) executive functions, via phonemic fluency (50, 51), overlapping pictures (52), and clock drawing (53).

#### 2.2.4 Statistical analyses of behavioral data

We investigated group differences in LA while considering different variables that might modulate punishment sensitivity in decision-making, i.e., age, education level, smoking status, global cognitive status, alongside attentional and executive performance (17), as well as sex (54). Since all these variables were normally distributed (Kolmogorov-Smirnov test, p>0.05), we used a) Pearson’s correlation to investigate their mutual relationship; b) a two-sample t-test to assess a differential LA across AUD patients and HCs; and c) an analysis of covariance (ANCOVA) to assess this difference while controlling for the aforementioned variables; d) a 2 x 2 (group x sex) factorial ANOVA to investigate whether LA was modulated by group, sex or their interaction. The statistical threshold was set at p<0.05, with False Discovery Rate (FDR; 55) correction applied within each family of behavioral tests.

In AUD patients, we additionally examined a possible association between LA and variables concerning: a) cognitive performance; b) alcohol use (disease duration and alcohol consumption) and global clinical status (BMI, nutritional and sleep status, anxiety and depression). Except for BMI, structured clinical interviews allowed to recode the latter variables in terms of discrete scores, with higher scores denoting worse conditions (**Supplementary Table 1**).

### 2.3 FDG-PET imaging

#### 2.3.1 Acquisition and reconstruction of 18F-FDG-PET data

AUD patients underwent a PET scan acquisition (GE Discovery 690 PET), lasting approximately 15 minutes, that was performed following the guidelines of the European Association of Nuclear Medicine (56). We collected static emission images 45 minutes after injecting 2,5 MBq/Kg of 18F-FDG in fasting patients. This post-injection time interval enables an equal distribution of the tracer across the entire brain, with negligible blood flow-dependent differences, and therefore an optimal signal-to-noise ratio (57). We used uniform reconstruction protocols, including both ordered subset-expectation maximization algorithm and CT attenuation correction procedures. A quality control process was performed to check for major artefacts in PET raw images, including defective image uniformity and orientation, or attenuation correction due to a mismatch between CT and PET images.

#### 2.3.2 Spatial pre-processing of 18F-FDG-PET images

We used the Statistical Parametrical Mapping (SPM12) software (https://www.fil.ion.ucl.ac.uk/spm/software/spm12/), as implemented in MATLAB 2021b (Mathworks Inc., Sherborn, Mass., USA), to perform a standard pre-processing of PET scans. Each image was first normalized to an 18F-FDG-PET template registered to the Montreal Neurological Institute (MNI) standard space (57) using the default SPM12 bounding-box, resampled at an isotropic voxel size of 2 mm, and spatially smoothed with an 8 mm isotropic 3D Gaussian Full-Width-Hald-Maximum (FWHM) kernel. The 18F-FDG-PET template has been reported to ensure high normalization accuracy, while reducing noise-related random effects (57, 58). Each image was proportionally scaled to its global mean (59, 60) to generate standardized uptake value ratio (SUVR) images and thus overcome issues of between-subject uptake variability (61). This approach enables higher signal-to-noise ratio compared to other available scaling methods (e.g., cerebellar reference area; 62). The same pre-processing pipeline was applied to 42 PET scans of age- and sex-matched healthy controls (henceforth “PET-HCs”), extracted from a larger, previously validated (57), dataset of 18F-FDG-PET of healthy individuals.

#### 2.3.3 Statistical analyses of 18F-FDG PET images

SPM12 was also used for statistical analyses, including: (1) a two-sample t-test, to assess the hypothesis of lower regional metabolism in AUD patients, compared with PET-HCs, while controlling for age (nuisance variable); (2) a multiple regression, in the patient sample, to assess a relationship between brain metabolism and LA in AUD, while controlling for disease duration; c) based on behavioral results (3.1), an interaction analysis, in the patient sample, to investigate a possible effect of sex on the relationship between brain metabolism and LA in AUD; d) a conjunction analysis, to assess the predicted anatomical overlap between the regions in which brain metabolism was both lower in patients (vs. PET-HCs), and related to LA. The statistical threshold was set at p<0.05, corrected for multiple comparisons with False discovery Rate (FDR; 55) at the cluster level (voxel-level forming threshold=p<0.001 uncorrected).

Based on our hypothesis and on behavioral (3.1) and neural (3.2) results, we used a follow-up moderation model to characterize the effect of sex on the relationship between LA and brain metabolism in the posterior frontomedial cortex, while controlling for disease duration. To this purpose, we first used the SPM toolbox Marsbar (http://marsbar.sourceforge.net/) to create a binary mask of the posterior frontomedial cluster showing the significant “sex X LA” interaction, and the toolbox Rex (https://www.nitrc.org/projects/rex/) to extract from this mask individual mean metabolism values. We then used the PROCESS macro (v.3.5) for SPSS (v.23, IBM, Armonk, NY, USA) to test Hayes’ (63) model 1 (moderation), through a conditional process analysis (64) based on Ordinary Least Squares (OLS) regression, using bootstrapping resampling (50,000 samples) to generate confidence intervals for direct and moderated effects. Interaction variables were mean-centered before entering the analyses, and the Johnson and Neyman’s (65) approach was used to compute the range of significance and simple slopes for the interaction analyses, which were assessed 1 SD below and above the mean, while applying a correction for heteroscedasticity. The statistical threshold was set at p<0.05 (two-tailed).

## 3 Results

### 3.1 Behavioral results

We found lower executive (overlapping figures) and attentional (TMT-A) performance in AUD patients compared with HCs, alongside a marginal trend for worse global cognitive performance and working-memory (interference memory-10’’) (**Supplementary Table 2**). However, none of these variables was significantly associated with LA (all p>0.05), which remained significantly lower in AUD patients (mean=1.90, SD = 0.23) than in HCs (mean=2.13, SD = 0.38) after adjusting for the potential effect of age, education level and smoking status (ANCOVA: F(1)=10.01, p=0.003). A formal *post-hoc* power analysis showed that the group effect accounted for partial η^2^ = 0.142 (Cohen’s f = 0.406), with an achieved *post-hoc* power of approximately 0.66. The lower LA in AUD patients remained significant also when excluding one potential outlier subject (ANCOVA: F(1)=8.159, p=0.008), and was confirmed by a 2x2 ANOVA (F(1)=4.992, p=0.032), which additionally showed a main effect of sex (F(1)=4.110, p=0.05), with females displaying larger LA than males, but no group-by-sex interaction (F(1)=0.017, p=0.896). In the patient sample, LA was not significantly related to alcohol use or clinical variables (all p>0.05).

### 3.2 FDG-PET results

**Figure 1:**
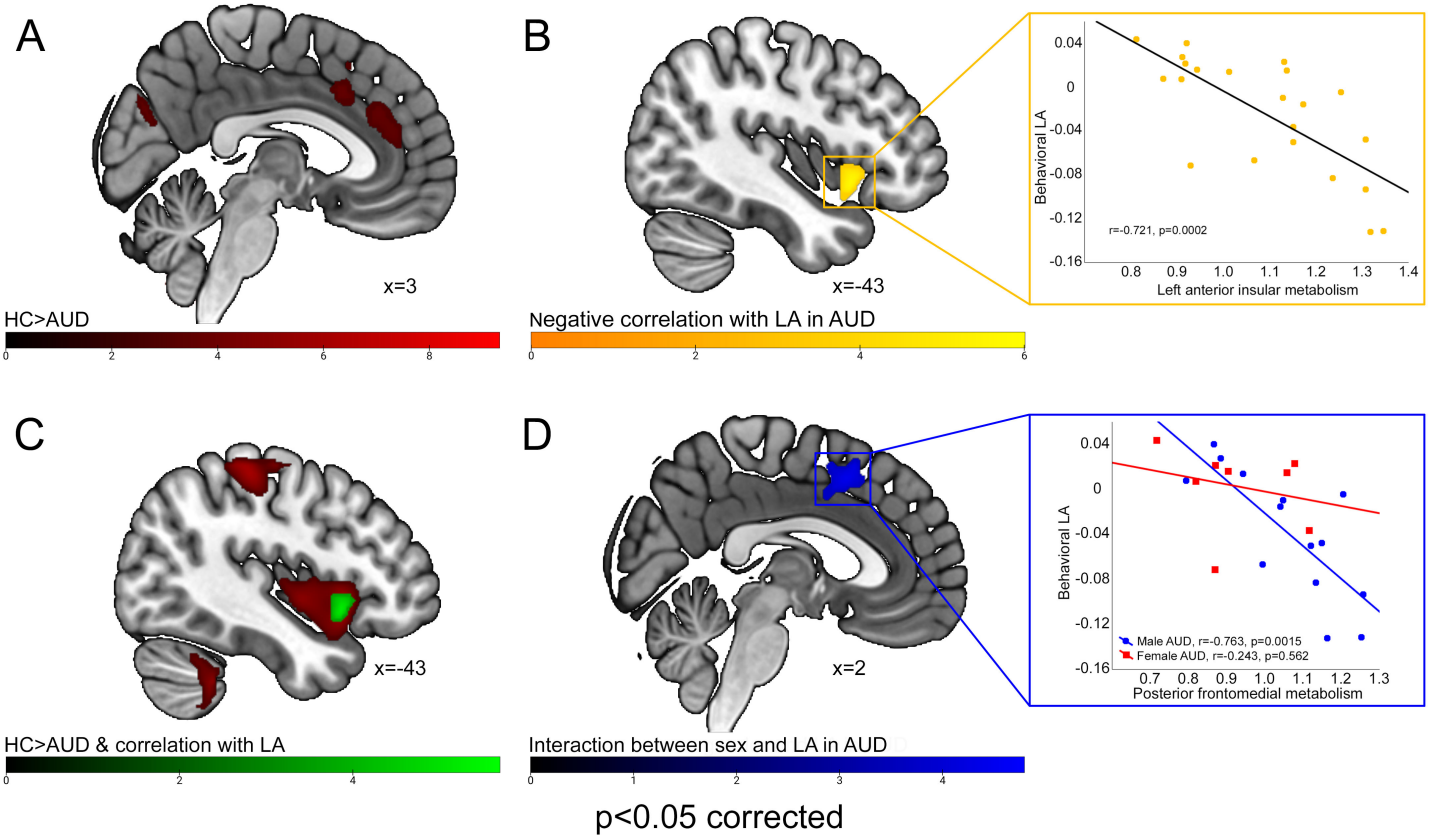
FDG-PET results. The figure reports the brain areas in which metabolism was **(A)** significantly lower in AUD patients compared with PET-HCs (red); **(B)** negatively correlated with behavioral LA in AUD patients (yellow); **(C)** both lower in AUD patients compared with PET-HCs and negatively correlated with behavioral LA (green); **(D)** more negatively correlated with behavioral LA in male than female AUD patients (blue).

Brain metabolism was significantly lower in AUD patients, compared with PET-HCs, in the posterior frontomedial cortex (including the anterior and midcingulate cortex), left fronto-insular cortex (extending into the rolandic and parietal opercula) and right temporopolar cortex. A bilateral hypometabolic pattern was found, in AUD patients, also in the posterior occipital cortex (inferior and middle occipital gyri), and sensorimotor cortex (**Figure 1**; **Table 1**). Instead, we observed higher metabolism, in AUD patients compared with PET-HCs, in a sector of the thalamus projecting to the prefrontal cortex, and in the posterior cingulate cortex (**Table 1**).

**Table 1:** FDG-PET results.

H	Brain region	Anatomy toolbox	x	y	z	T	K	p-corrected
a) HC > AUD (hypometabolism in AUD)								
L/R	Anterior cingulate cortex		2	36	28	4.09	374	**0.0348**
	Middle cingulate cortex		2	18	38	3.95		
	Posterior frontomedial cortex		2	20	50	3.51		
L	Anterior insular cortex		-40	16	-4	5.99	1449	**<0.0001**
	Parietal operculum	Area OP4 [PV]	-56	-10	6	4.6		
	Precentral Gyrus	Area 44	-54	4	20	4.18		
	Parietal operculum	Area OP1 [SII]	-60	-22	12	3.95		
	IFG (pars opercularis)	Area 44	-50	14	10	3.78		
L	Postcentral Gyrus	Are 3b	-38	-36	64	6.1	838	**0.0012**
	Postcentral Gyrus	Area 4a	-38	-34	60	5.99		
	Postcentral Gyrus	Area 1	-48	-30	58	5.87		
	Superior Parietal Lobule	Area 7A (SPL)	-30	-60	62	4.3		
R	Postcentral Gyrus	Area 1	42	-36	64	6.1	554	**0.0076**
	Precentral Gyrus		50	-8	48	3.91		
R	Middle Occipital Gyrus		30	-74	38	5.6	697	**0.0028**
	Cuneus		10	-78	32	5.16		
	Superior Occipital Gyrus		24	-80	22	3.35		
R	Cerebellum	Lobule VIIa Crus1	56	-64	-34	6.07	2589	**<0.0001**
L	Cerebellum	Lobule VIIa Crus1	-40	-44	-42	5.65		
b) AUD > HC (hypermetabolism in AUD)								
R	Thalamus	Thal: prefrontal	12	-12	8	8.70	225	**p<0.0001**
L	Thalamus	Thal: prefrontal	-16	-26	8	7.80	180	**p<0.001**
L/R	Posterior cingulate cortex		-6	-54	16	9.62	608	**p<0.0001**
c) Negative correlation between brain metabolism and behavioral LA in AUD									
L	Anterior insular cortex		-44	14	-8	6.07	144	**0.025**
d) Common effects of a) and c)									
L	Anterior insular cortex		-44	14	-10	5.76	101	**0.023**
e) Interaction between sex and behavioral LA on brain metabolism in AUD									
R	Posterior frontomedial cortex		4	14	48	4.86	206	**0.023**
L/R	Middle cingulate cortex		-2	4	40	4.40		
L/R	Posterior frontomedial cortex		0	6	54	3.89		

From top to bottom, the table reports the regions in which brain metabolism was a) significantly lower in AUD patients vs. PETHCs; b) significantly higher in AUD patients vs. PET-HCs; c) negatively correlated with behavioral LA in AUD patients; d) both significantly lower in AUD patients vs. PET-HCs and negatively correlated with behavioral LA in AUD patients; e) modulated by the interaction between sex and behavioral LA.

A negative correlation between behavioral LA and brain metabolism was found, in AUD patients, in the left ventral anterior insula, in which a conjunction analysis confirmed a hypometabolic pattern (**Figure 1**; **Table 1**).

An interaction analysis additionally showed that this relationship was significantly modulated by sex in the posterior frontomedial cortex (**Figure 1**; **Table 1**). To characterize this effect, a conditional process analysis yielded a significant model (F(4,17)=9.1867, p=0.0004; R^2^ = .6001) in which behavioral LA was inversely predicted by frontomedial metabolism (p=0.0027) and by its interaction with sex (F(1,17)=6.1717, p=0.0237), whereas sex showed no significant main effect (p=0.1350) (**Table 2**). This interaction reflects the significant negative correlation between behavioral LA and frontomedial metabolism observed in male (r=-0.7630, p=0.002), but not female (r=-0.2428, p=0.562), AUD patients.

**Table 2:** Interaction “sex X posterior frontomedial metabolism” on LA.

Model summary						
R	R2	MSE	F	DF1	DF2	p
0.7746	0.6001	0.0014	9.1867	4	17	0.0004
Model						
	Coeff	SE	t	p	LLCI	ULCI
Posterior frontomedial metabolism	-0.5211	0.1486	-3.5067	0.0027	-0.8346	-0.2077
Sex	0.0225	0.0143	1.5734	0.135	-0.0078	0.0528
Interaction sex x posterior frontomedial metabolism	0.2277	0.0917	2.4831	0.0237	0.0343	0.4212
Disease duration	0.0017	0.001	1.7000	0.1075	-0.0004	0.0039
Test(s) of highest order unconditional interaction(s)						
	R2-change	F	DF1	DF2	p	
Sex x posterior frontomedial metabolism	0.0805	6.1717	1	17	0.0237	
Conditional effects of posterior frontomedial metabolism at values of sex						
Moderator “sex”	Effect	SE	t	p	LLCI	ULCI
Sex=male	-0.2934	0.0674	-4.3519	0.0004	-0.4357	-0.1511
Sex=female	-0.0657	0.0618	-1.0615	0.3033	-0.1962	0.0648
Bootstrap results for regression model parameters						
	Coeff	BootMean	BootSe	BootLLCI	BootULCI	
Posterior frontomedial metabolism	-0.5211	-0.5990	0.1898	-0.9644	-0.1918	
Sex	0.0225	0.0282	0.0270	-0.0162	0.0547	
Interaction sex x posterior frontomedial metabolism	0.2277	0.2939	0.1893	0.0060	0.5530	
Disease duration	0.0017	0.0017	0.0014	-0.0011	0.0046	

Conditional process analysis. The table reports the coefficients and 95% confidence intervals of moderation for predicting behavioral LA based on AUD patients’ sex and posterior frontomedial metabolism.

MSE: Mean Squared Error; DF: degrees of freedom; LLCI: lower level of confidence interval; ULCI: upper level of confidence interval; coeff: coefficient; se: standard error. Bold font denotes p<0.05.

A formal *post-hoc* power analysis of this sex-by-metabolism interaction showed that the interaction term accounted for an incremental ΔR^2^ = 0.0805, corresponding to Cohen’s f^2^ = 0.201. At the observed sample size (n=22), the achieved *post-hoc* power was approximately 0.51.

H: hemisphere; HC: healthy controls; AUD: Alcohol Use Disorder; L: left; R: right; OP: parietal operculum; SII: secondary somatosensory cortex; IFG: inferior frontal gyrus; SPL: superior parietal lobule; K: cluster extent in number of voxels (2x2x2 mm^3^). Bold font denotes a statistically significant effect at p<0.05 corrected for multiple comparisons.

## 4 Discussion

We used a mixed-gamble task (66) to investigate the neural basis of altered LA in 22 AUD patients - who also underwent 18F-FDG-PET - compared with 19 age-, sex- and education-matched healthy controls. Consistent with previous evidence (17, 18), LA was lower in patients than in controls. Although the behavioral data additionally showed a main effect of sex, the PET findings specifically suggested that the neural correlates of LA may be differentially modulated by sex within AUD. In particular, lower LA was associated with a steeper negative relationship with frontomedial metabolism in male, compared with female, AUD patients.

In keeping with previous evidence from resting-state fMRI (67), compared with PET-HCs patients displayed a significant hypometabolism in key nodes of the salience network such as the dorsal anterior cingulate and left fronto-insular cortex (68). As previously suggested (69), this pattern may help explain their executive impairments in terms of a defective switch from automatic to controlled cognitive processes. However, the present evidence of lower LA in AUD patients could not be explained by their impaired attentional or working-memory performance. This finding suggests that altered susceptibility to punishment may reflect, in AUD, a neural mechanism specific to evaluation and decision-making, possibly involving the interoceptive boost to punishment avoidance (27) and/or conflict resolution at the response level (18). Consistent with this, we found evidence of hypometabolism in the left anterior insula in AUD compared with PET-HCs. The anterior insula, particularly in the left hemisphere, has been previously associated with processing emotional stimuli (70, 71) and their negative valence ratings (72), as well as pre-response conflict (73) and post-outcome behavioral adjustment (74). These findings have strengthened the view that the insula might contribute to decision-making (75), particularly in association with risky choices (76), by coding the salience of adverse outcomes (77). The observed hypometabolism of the anterior insula might thus be associated with reduced salience of losses and lower loss avoidance in AUD patients.

However, when focusing on AUD patients, we observed a negative correlation between left insular metabolism and LA (i.e., lower metabolism reflects higher LA), which calls for an additional mechanism to explain why, within the AUD sample, the lowest insular metabolism was associated with the highest LA. Given the baseline hypometabolism observed in the AUD group compared to PET-HCs, this unexpected association, linking the lowest metabolic rates to the highest LA, requires a distinct explanatory model. The available evidence suggests that specific neural processes may drive this divergent pattern, independent of the general metabolic deficit. Resting-state fMRI studies reported altered functional connectivity of the left anterior insula in AUD (78–82). In particular, graph-theoretical measures showed higher centrality of this region, suggestive of stronger integration of intra- and inter-network connections. This observation is consistent with reports of larger insular connectivity in AUD (81, 83, 84) and in a rat model of AUD (85) and suggests that the anterior insula may promote hyper-integration of interoceptive states in early abstinence, thereby influencing emotional states and decision-making (78). This mechanism might interact with poorer regulation of negative emotions in AUD (86, 87), again linked to lower insular activity (88; see 89). Together, such processes of hyper-integration of interoceptive states and altered emotion regulation may help explain why, within the AUD sample, the lowest insular metabolism was associated with the highest LA. Compared with PET-HCs, AUD patients showed significantly reduced metabolism in the salience network. This finding may be related to a diminished salience of losses and reduced loss avoidance in AUD, consistent with the behavioural results showing lower loss aversion in AUD than in HCs.

More cautiously, the finding that the negative relationship between behavioral LA and frontomedial metabolism was significant only in male AUD patients suggests a possible sex-related modulation of this circuitry. The posterior frontomedial cortex has been associated with response conflict (90), inhibition (91), and impulsivity (92), and the latter is considered a particularly relevant risk factor for problem drinking in men (31).

A notable limitation of this study is the relatively small sample size, particularly for sex-disaggregated analyses, which limits strong conclusions about the observed sex differences, as these may partly reflect unmeasured or insufficiently controlled confounders rather than sex *per se*. This consideration is particularly relevant for the moderation analysis of the relationship between loss aversion and posterior frontomedial metabolism. Although supported by a not negligible effect size and by bootstrap confidence intervals, this analysis should still be regarded as underpowered for a definitive conclusion, and the observed sex-related modulation is best interpreted as preliminary. Moreover, although smoking status did not differ significantly between AUD patients and HCs, its unequal distribution across groups may still have introduced residual confounding, given the potential impact of nicotine exposure on both behavior and brain function. Importantly, however, smoking status was not significantly associated with LA, which remained significantly lower in AUD patients than in HCs after adjusting for its potential effect alongside age and education level. The PET analysis was limited by the use of an external control dataset. While the behavioral HC group was controlled for several relevant factors (see **Supplementary Materials**), the PET-HC group was matched only for age and sex. Information on key determinants of brain metabolism, including smoking status, BMI, nutrition, sleep, and anxiety was unavailable for the external PET-HCs, thus constraining the interpretation of group differences. Finally, despite enrolment after 28 days of withdrawal treatment and at least 10 days of detoxification, metabolic and cognitive measures may still reflect clinical factors related to early abstinence. Follow-up studies are thus needed to establish whether these findings represent stable features of lifetime AUD.

Despite these limitations and the need for follow-up studies, our findings offer novel insights into the hypometabolic brain patterns associated with lower LA in AUD and suggest that their neural correlates may be differentially modulated by sex. In line with the behavioral, cognitive and affective expression of AUD, the present evidence supports the view that, in this condition, abnormal processing of negative vs. positive choice consequences involves key brain nodes of emotion regulation (93), interoception (94) and attentional processing (95). These regions may represent candidate targets for future rehabilitation studies, including non-invasive brain stimulation approaches (96) and pharmacotherapies (97), but such translational implications remain preliminary and require dedicated testing. Observed sex-related modulation may likewise inform future research aimed at clarifying whether different neuro-pathological mechanisms contribute to AUD in females vs. males.

## Data Availability

The raw data supporting the conclusions of this article will be made available by the authors, without undue reservation.
