# Supplementary material for: Neural correlates of altered loss aversion in alcohol use disorder: preliminary evidence of sex-related differences from 18F-FDG-PET imaging
Source: Front Psychiatry. 2026 May 13;17:1847372. doi: 10.3389/fpsyt.2026.1847372 (PMC13212444; doi:10.3389/fpsyt.2026.1847372)
Supplement: Supplementary file 1 [file DataSheet1.pdf]

Supplementary Materials

Supplementary Table 1. Demographics and alcohol use variables.

Demographic variables (HC and AUD)	Age (years)	Education (years)	Sex (females/males)	Smoking status (yes/no)
HC: Mean (SD)	45.10 (8.69)	10.21 (2.74)	8/11	6/13
AUD: Mean (SD)	46.31 (7.99)	9.90 (2.65)	9/13	14/8
FDR p-value	0.803	0.803	0.810	0.082
Alcohol use variables (AUD only)	Duration of alcohol use (years)	Average daily alcohol intake (UA)	Abstinence before MRI (days)	Past use of other substances
All patients	10.11 (6.57)	14.34 (6.66)	17.13 (13.78)	
Females: Mean (SD)	10.17 (7.44)	13.53 (7.23)	14.37 (5.37)	None
Males: Mean (SD)	10.00 (5.18)	15.75 (5.70)	18.71 (16.84)	Marijuana (n=1); cocaine and marijuana (n=2)
FDR p-value	0.952	0.700	0.700	
Clinical parameters (AUD only)	BMI	Nutritional status	Sleep status	Anxiety level
All patients	23.90 (4.54)	0.86 (0.94)	1.63 (1.00)	1.04 (1.04)
Females: Mean (SD)	22.27 (2.88)	1.12 (0.99)	2.00 (0.75)	1.62 (1.06)
Males: Mean (SD)	24.82 (5.13)	0.71 (0.91)	1.42 (1.08)	0.71 (0.91)
FDR p-value	0.530	0.672	0.530	0.230

The top table section reports the mean and standard deviation (SD) of demographic variables and smoking status for alcoholic patients (AUD) and healthy controls (HC), alongside the results of group comparisons. The bottom sections report, for the whole patient sample and separately for females and males, alcohol use variables and distinct clinical parameters: body-mass index (BMI), nutritional status in terms of appetite (0=intense, 1=normal, 2=poor), sleep status (0=restful, 1=normal, 2=irregular, 3=disturbed) and anxiety level (0=none, 1=low, 2=moderate, 3=high). No patient displayed symptoms of depression. UA: Units of Alcohol, SD: standard deviation.

Supplementary Table 2. Neuro-cognitive performance.

Group differences in cognitive abilities (two-sample t-test)						
Variable	Mean (SD) HC	Mean (SD) AUD	DF	T-score	p-value	FDR p-value
**Global cognitive score**	84.10 (7.18)	78.63 (8.71)	39	2.170	0.036	**0.053**
**Interference memory 10"**	7.57 (1.57)	6.36 (1.98)	39	2.144	0.038	**0.053**
Interference memory 30"	6.94 (1.95)	6.68 (2.25)	39	0.399	0.691	0.691
**Trail-Making test A**	19.47 (5.46)	28.90 (6.48)	39	-4.991	<0.0001	**<0.0001**
Trail-Making test B	69.15 (21.33)	89.81 (42.59)	39	-1.914	0.062	0.073
**Overlapping figures**	36.94 (5.35)	31.54 (5.28)	39	3.244	0.002	**0.008**

For each neuro-cognitive variable, the mean and standard deviation (SD) are reported for healthy controls (HC) and alcoholic patients (AUD), alongside the results of group comparisons. Bold font denotes a statistically significant effect after correcting for multiple tests. DF: degrees of freedom; FDR: False Discovery Rate.
